# Supplementary figures and images for: CYP20-3 deglutathionylates 2-CysPRX A and suppresses peroxide detoxification during heat stress
Source: Life Sci Alliance. 2020 Jul 30;3(9):e202000775. doi: 10.26508/lsa.202000775 (PMC7409537; doi:10.26508/lsa.202000775)

**2CPA<sup>ox</sup>****2CPB<sup>ox</sup>****GSH****+****-****-****+****-****-****GSSG****-****+****-****-****+****-****GSNO****-****-****+****-****-****+**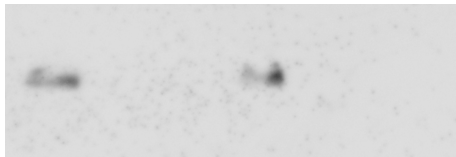**■ GSH-α**

Supplement: Supplementary file 1 [file LSA-2020-00775_SdataFS1.pdf]

**A**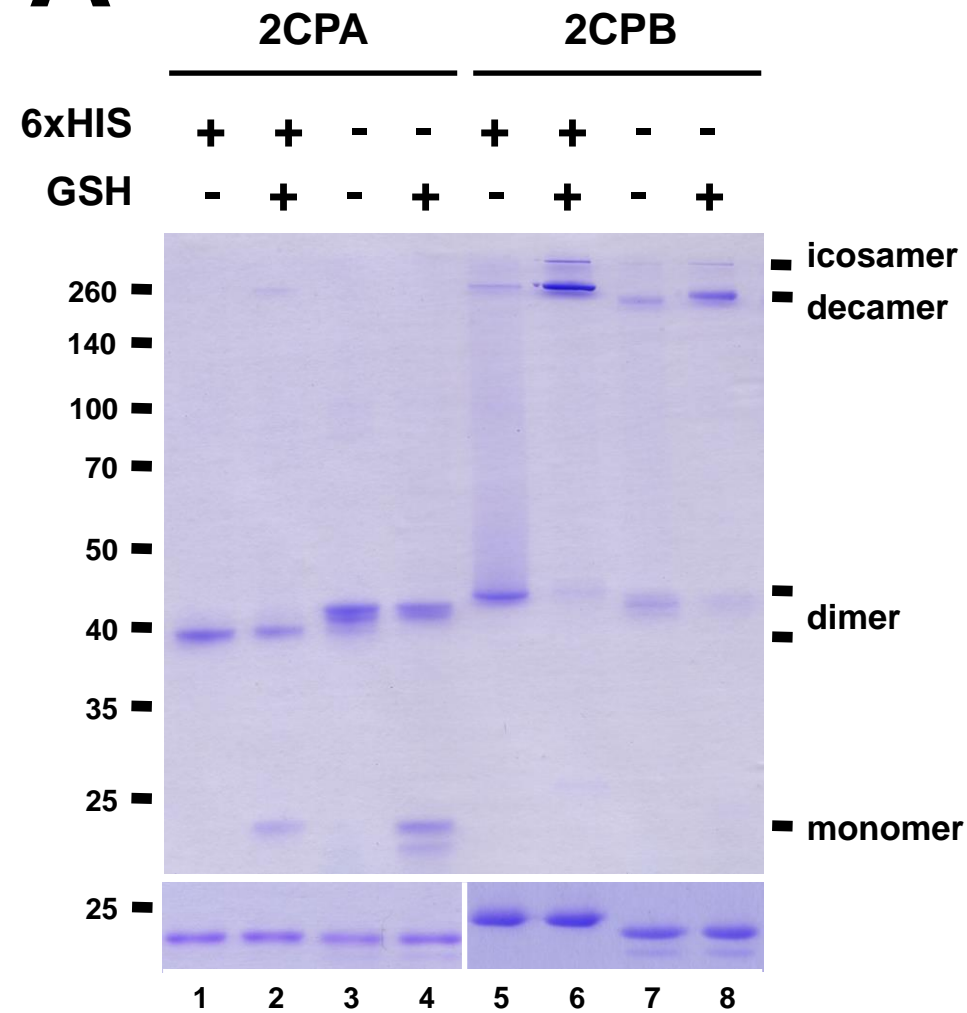**B**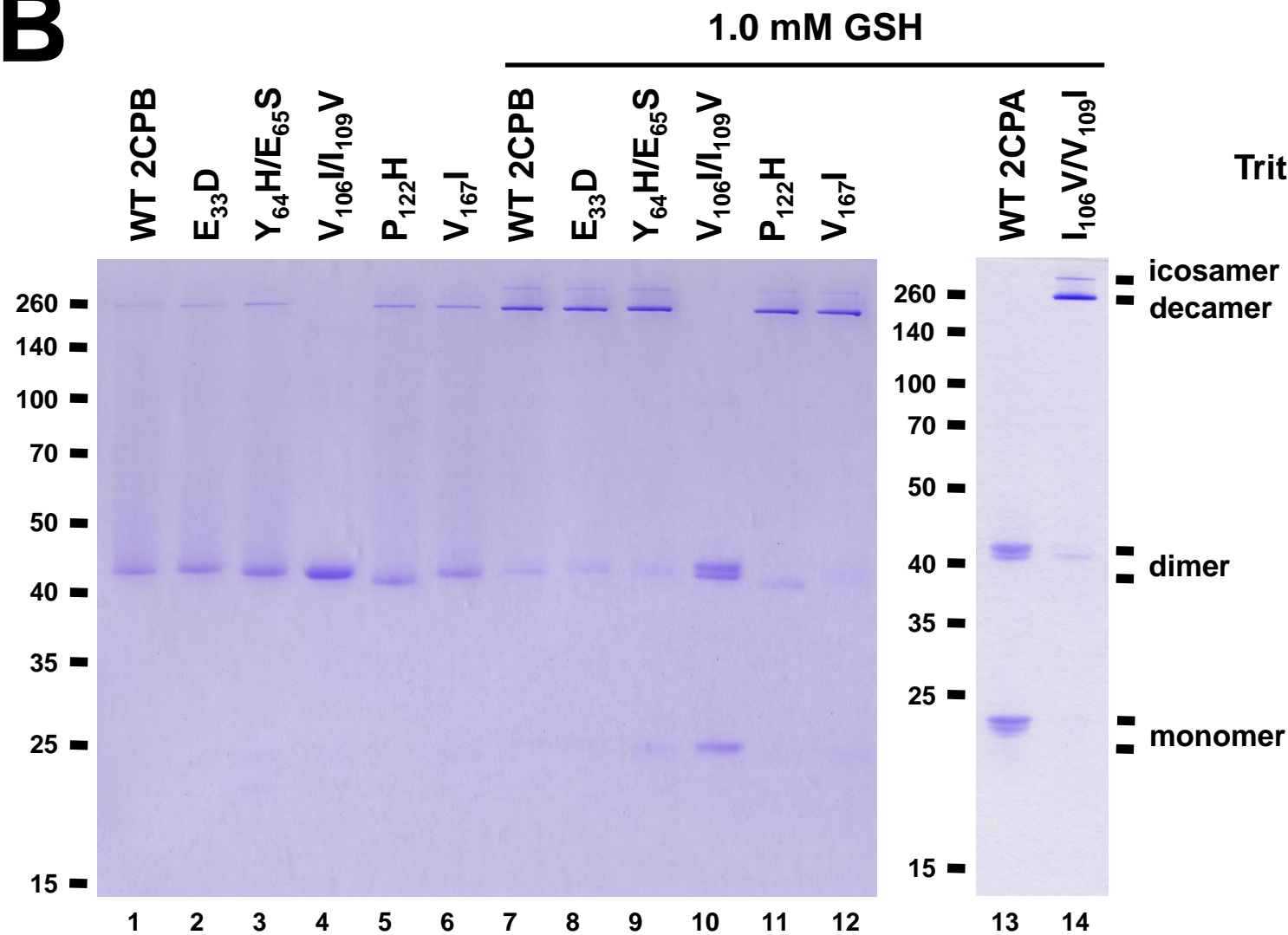**C**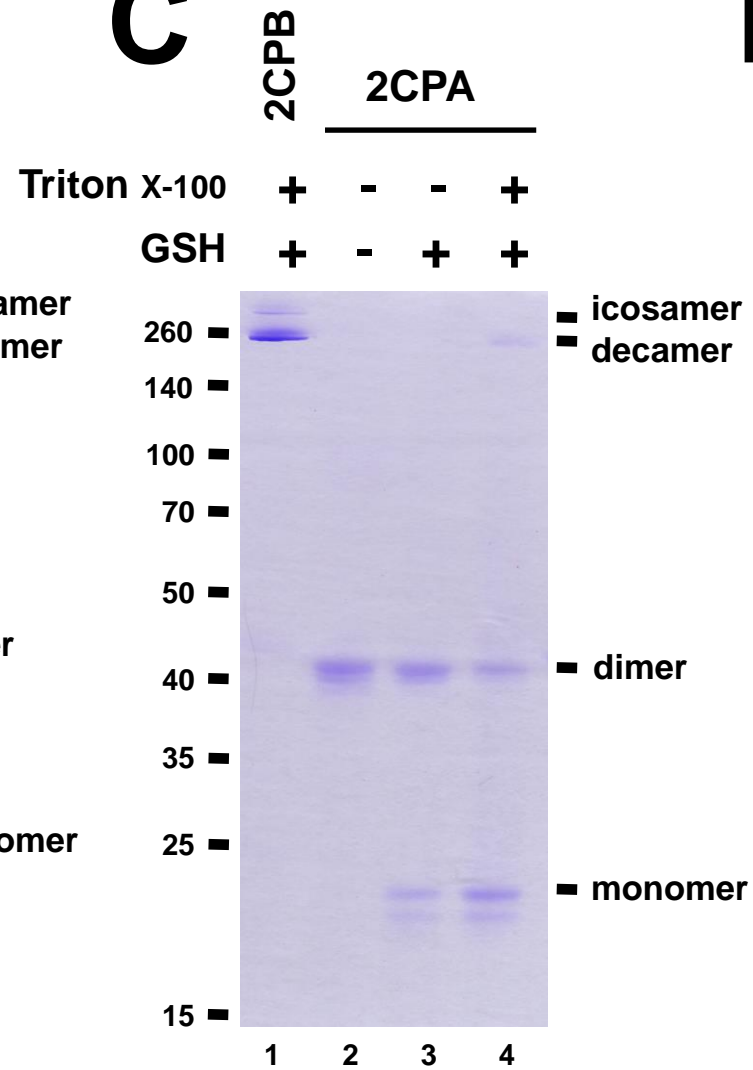**D**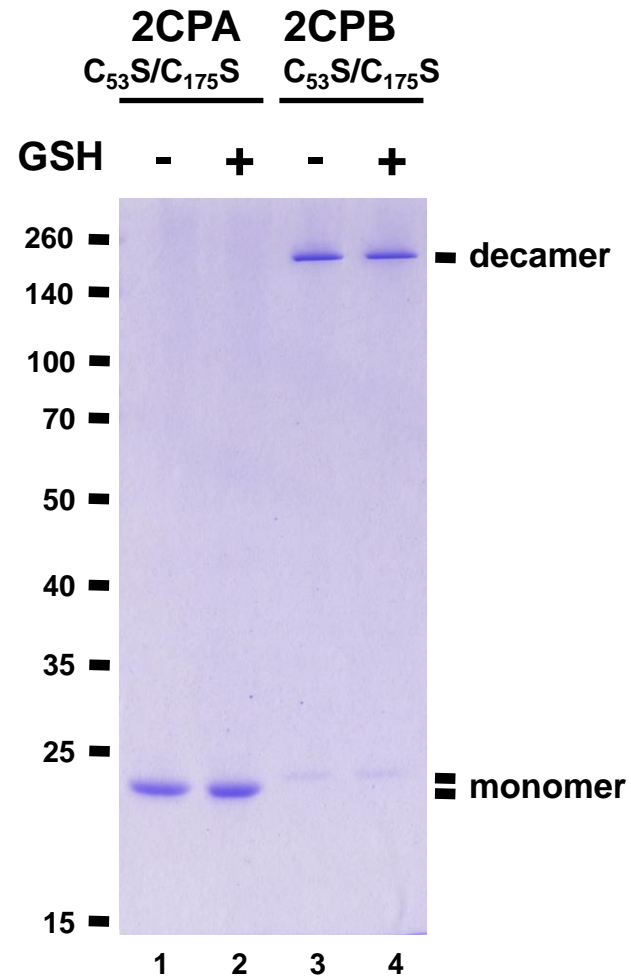

Supplement: Supplementary file 2 [file LSA-2020-00775_SdataF1.pdf]

**A**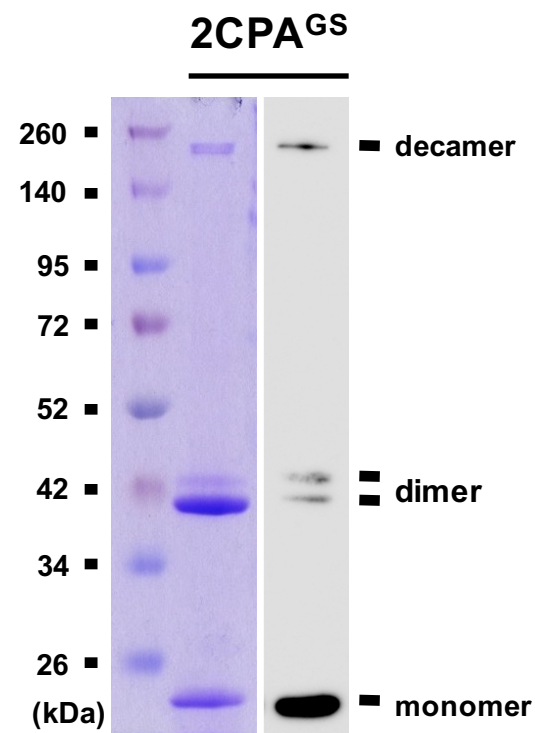**B**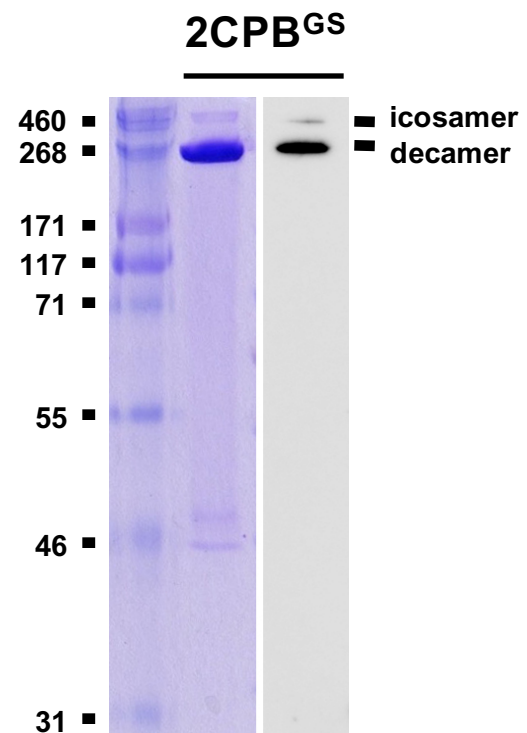**C**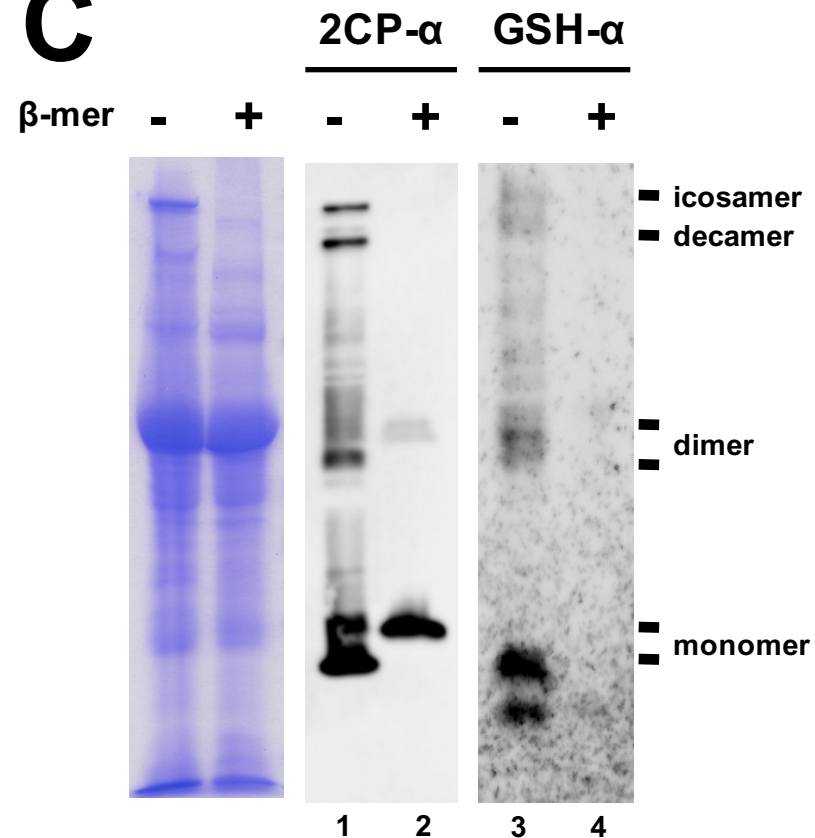**D**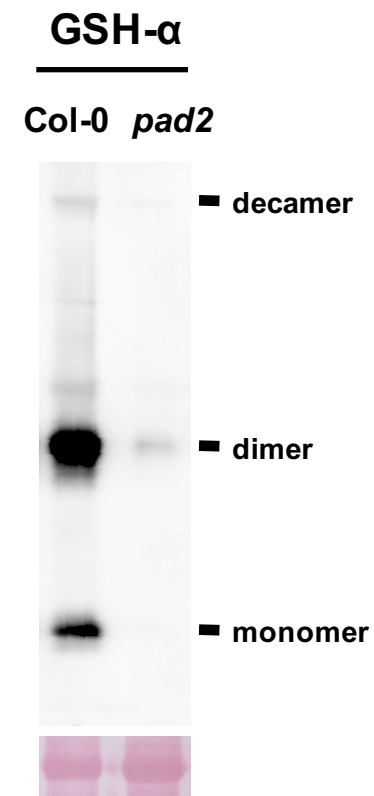

Supplement: Supplementary file 3 [file LSA-2020-00775_SdataFS2.pdf]

**A****GSH- $\alpha$** **2cpa 2cpb**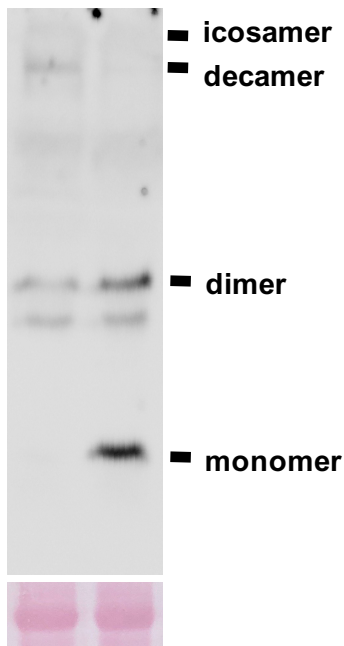**B**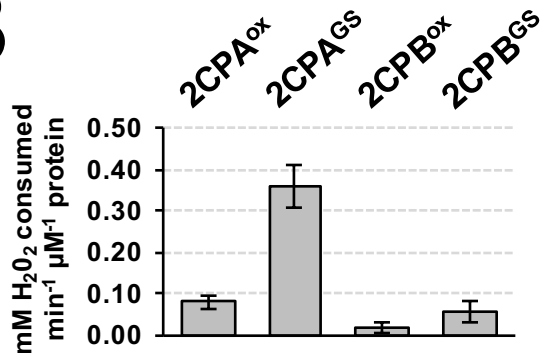**C**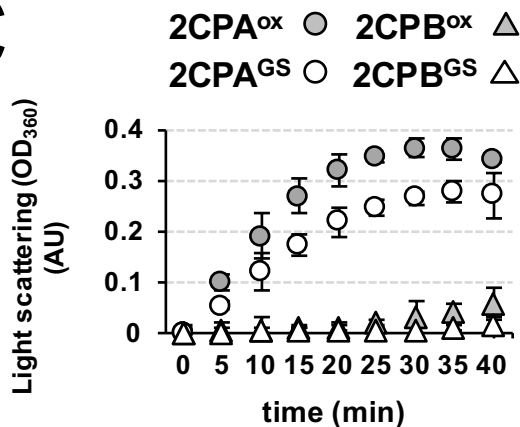

Supplement: Supplementary file 4 [file LSA-2020-00775_SdataFS3.pdf]

**2CP- $\alpha$**

---

|  | <i>cyp20-3</i> |  |  | Col-0 |  |  |
|--|----------------|--|--|-------|--|--|
|--|----------------|--|--|-------|--|--|

---

| hpw | 0 | 3 | 6 | 0 | 3 | 6 |
|-----|---|---|---|---|---|---|
|-----|---|---|---|---|---|---|

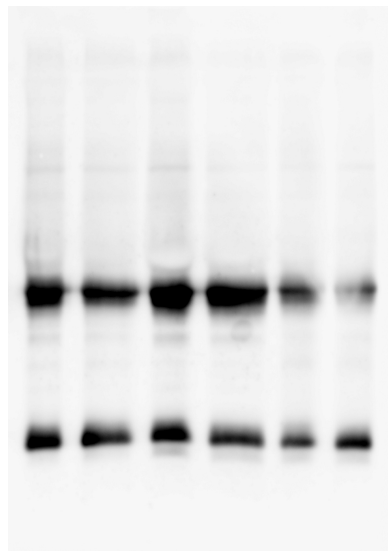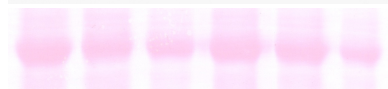

Higher  
Exposure

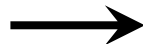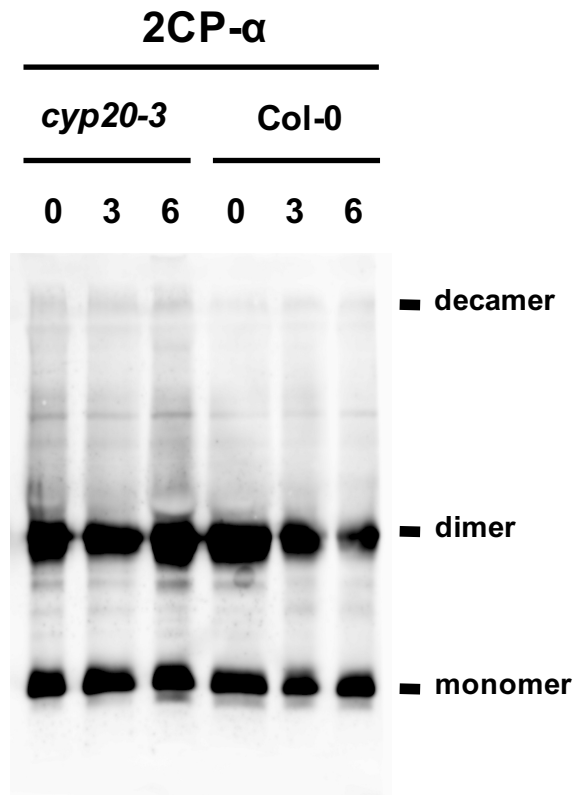

Supplement: Supplementary file 7 [file LSA-2020-00775_SdataFS5.pdf]

## 2CPA<sup>GS</sup>

500 (μM)    - NADPH   DTT

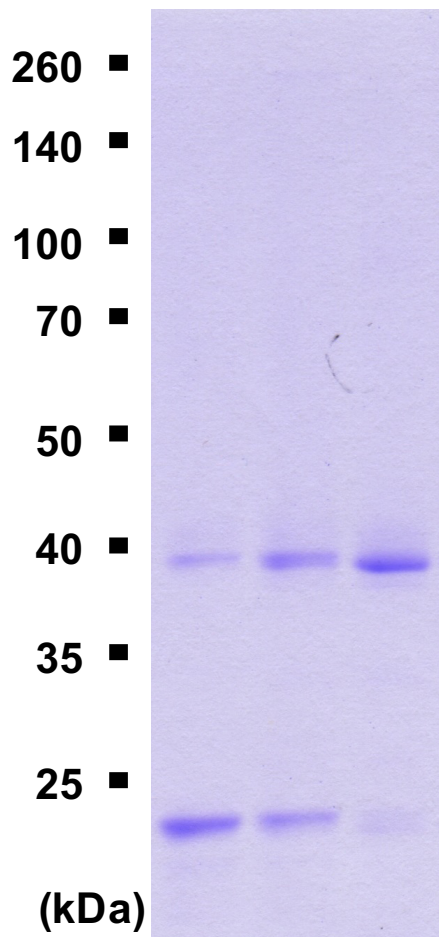

## 2CPB<sup>GS</sup>

- NADPH   DTT

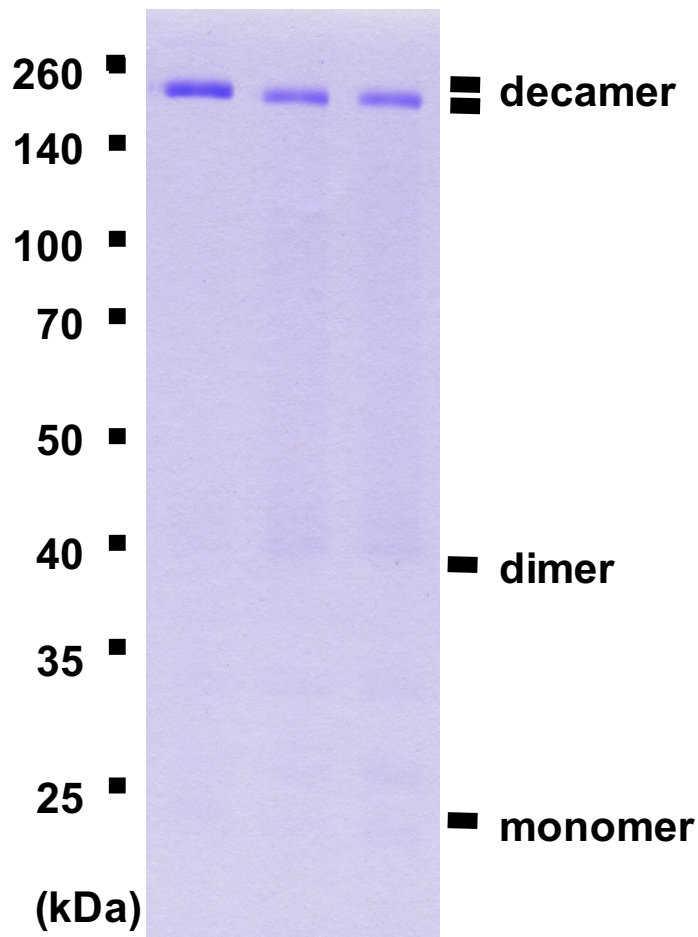

Supplement: Supplementary file 8 [file LSA-2020-00775_SdataFS6.pdf]

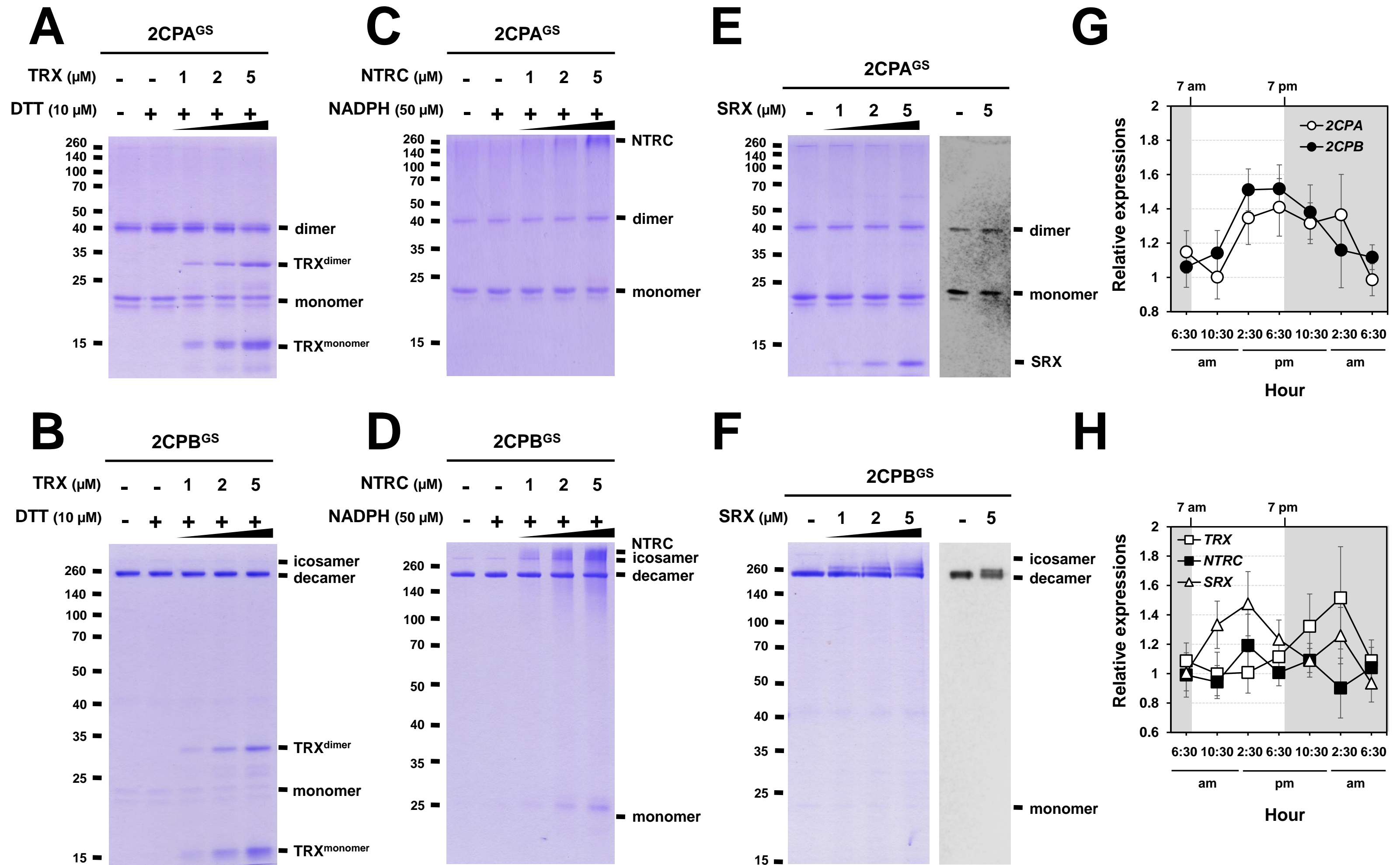

Supplement: Supplementary file 9 [file LSA-2020-00775_SdataF3.pdf]

## 2CPA<sup>GS</sup>

CYP20-3 (μM)

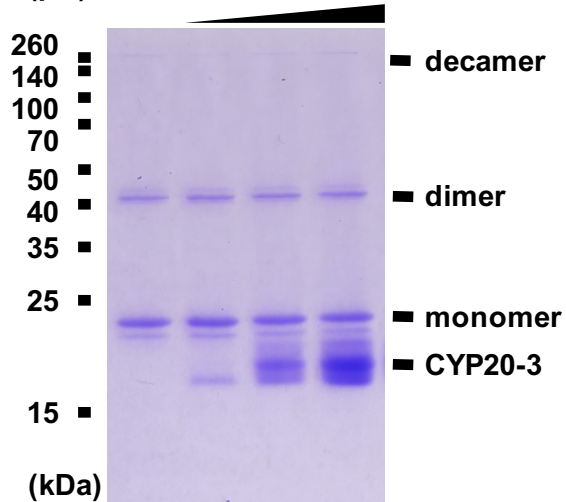

## 2CPB<sup>GS</sup>

CYP20-3 (μM)

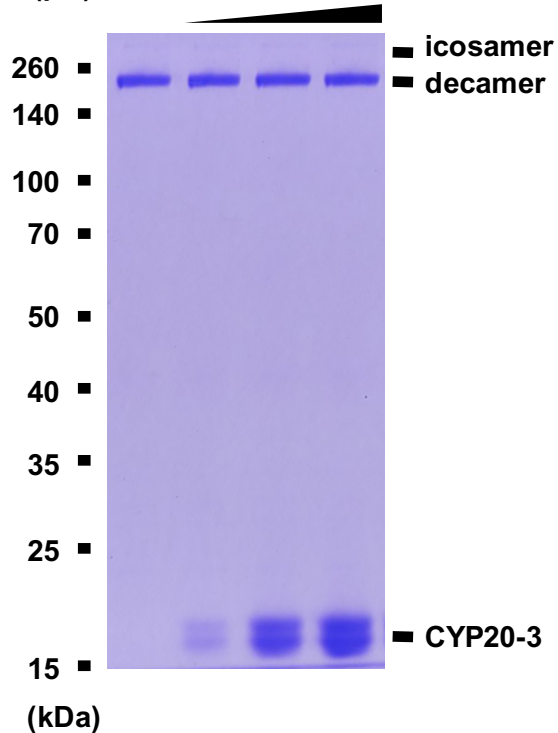

Supplement: Supplementary file 10 [file LSA-2020-00775_SdataFS7.pdf]

**A**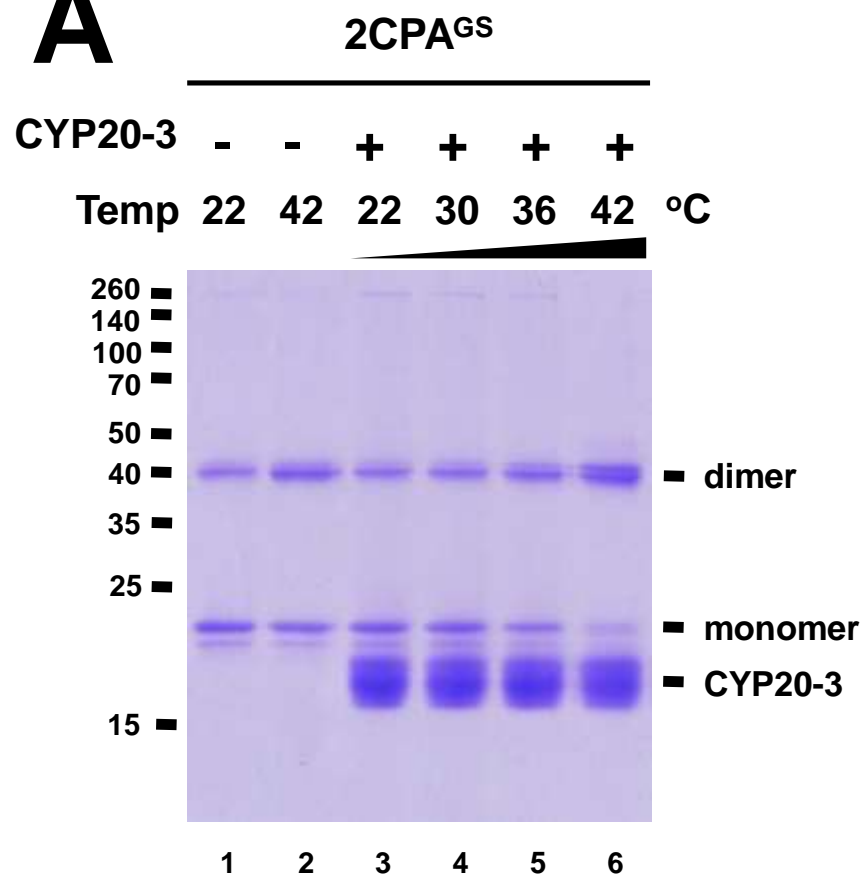**C**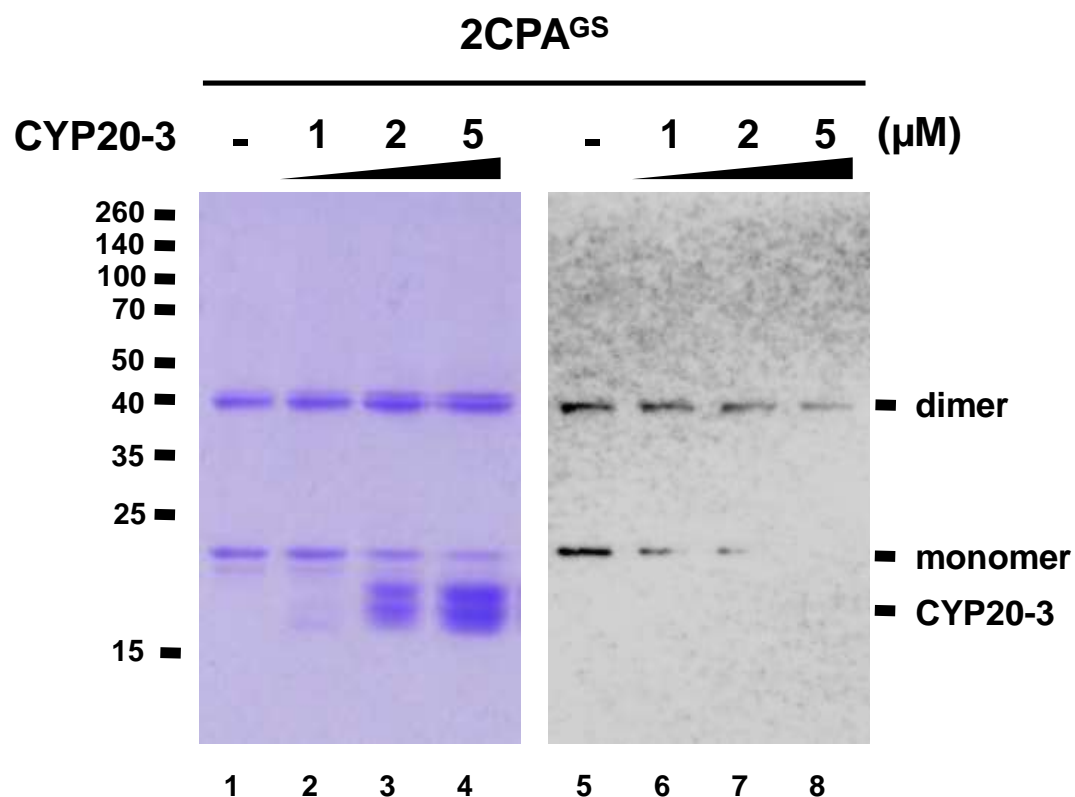**B**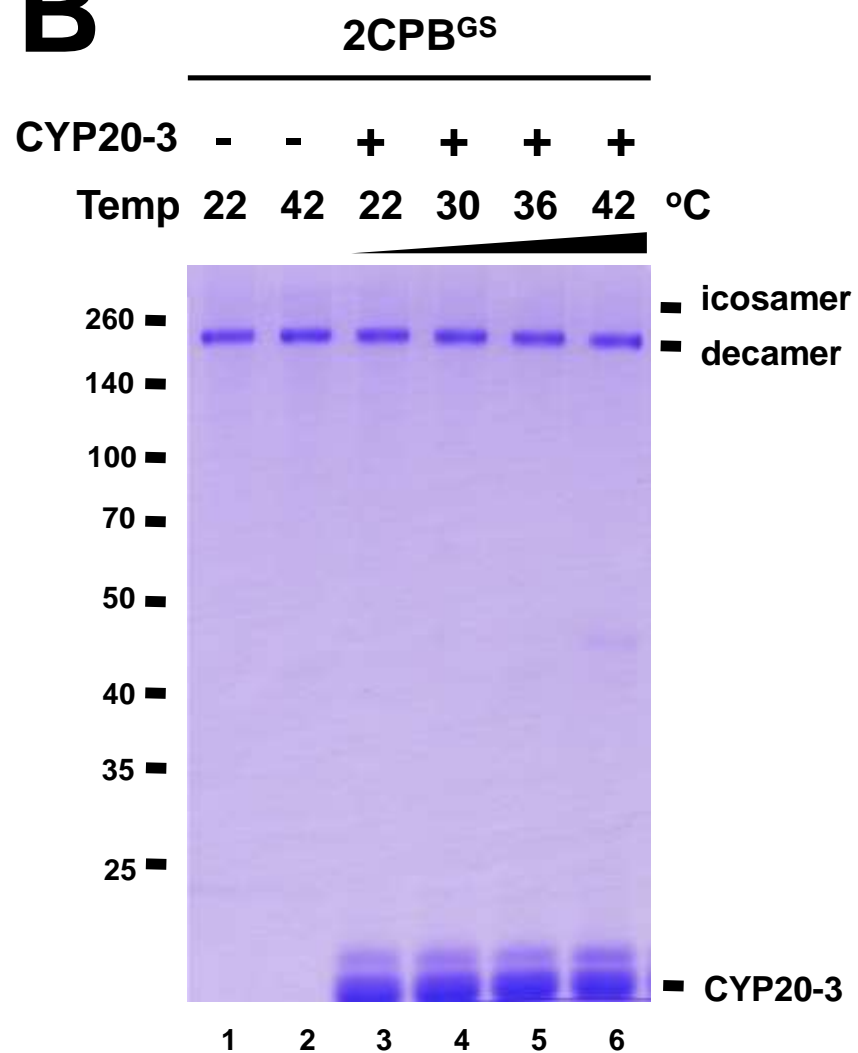**D**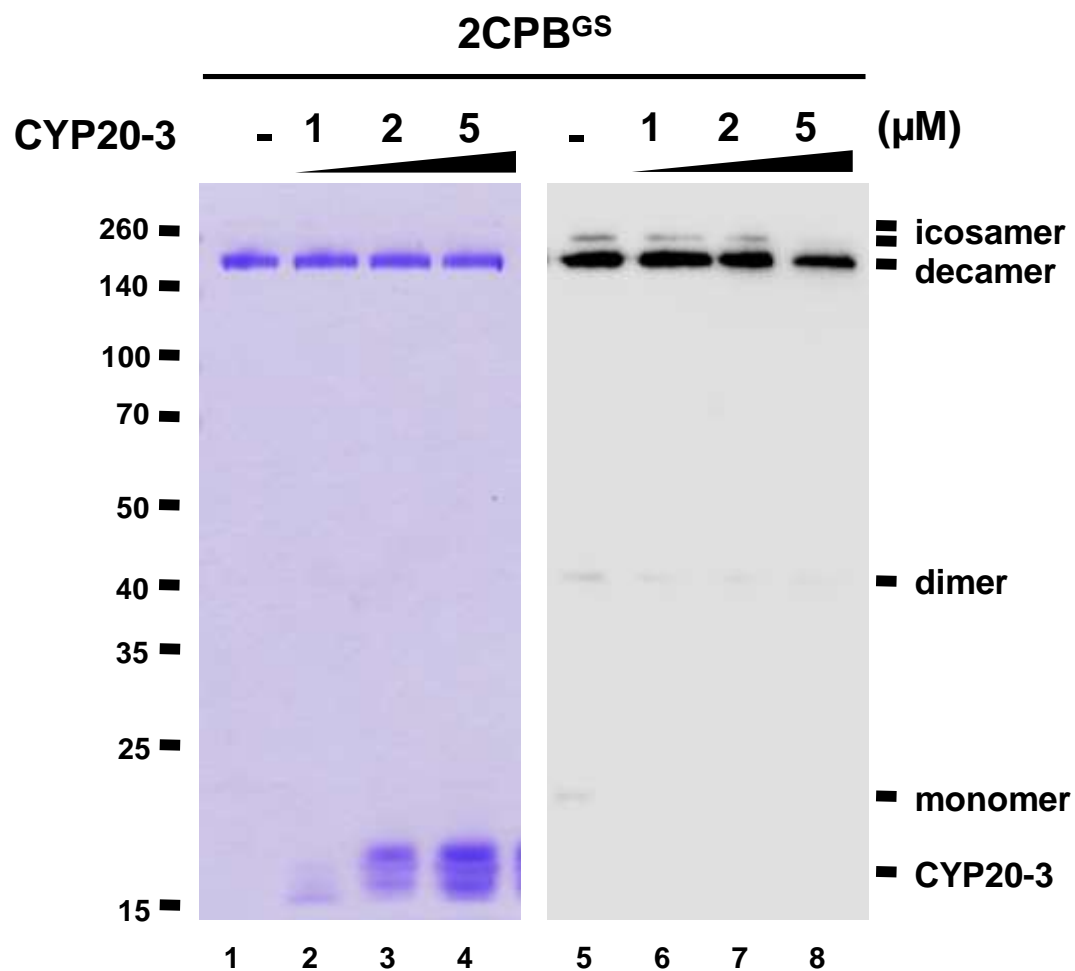

Supplement: Supplementary file 11 [file LSA-2020-00775_SdataF4.pdf]

# 2CPA<sup>GS</sup>

|               |   |   |   |   |   |
|---------------|---|---|---|---|---|
| NTRC (μM)     | - | - | 1 | 2 | 5 |
| NADPH (50 μM) | - | + | + | + | + |

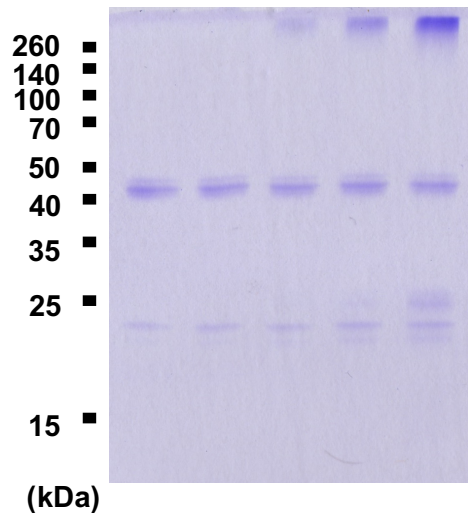

# 2CPB<sup>GS</sup>

|             |   |   |   |   |   |
|-------------|---|---|---|---|---|
| TRX (μM)    | - | - | 1 | 2 | 5 |
| DTT (10 μM) | - | + | + | + | + |

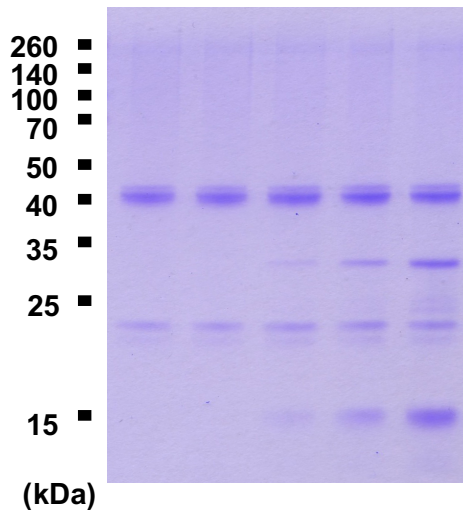

# 2CPA<sup>GS</sup>

|          |   |   |   |   |
|----------|---|---|---|---|
| SRX (μM) | - | 1 | 2 | 5 |
|----------|---|---|---|---|

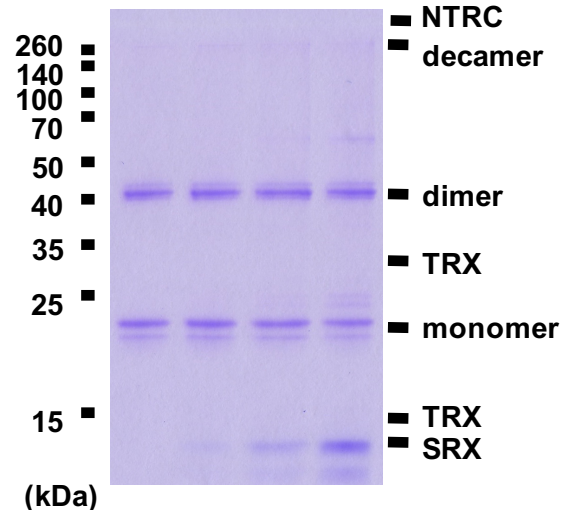

Supplement: Supplementary file 12 [file LSA-2020-00775_SdataFS8.pdf]

# 2CPA<sup>GS</sup>

| NTRC (μM)     | - | - | 1 | 2 | 5 |
|---------------|---|---|---|---|---|
| NADPH (50 μM) | - | + | + | + | + |

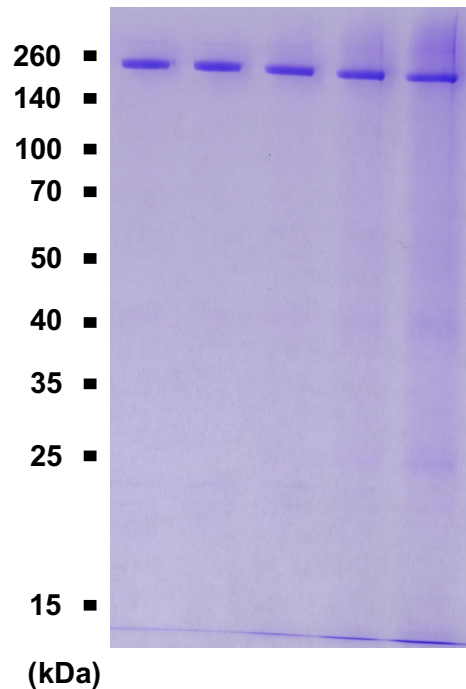

# 2CPB<sup>GS</sup>

| TRX (μM)    | - | - | 1 | 2 | 5 |
|-------------|---|---|---|---|---|
| DTT (10 μM) | - | + | + | + | + |

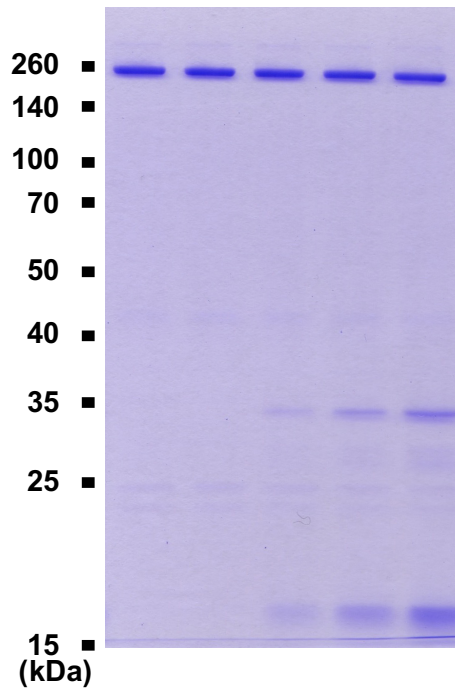

# 2CPA<sup>GS</sup>

| SRX (μM) | - | 1 | 2 | 5 |
|----------|---|---|---|---|
|----------|---|---|---|---|

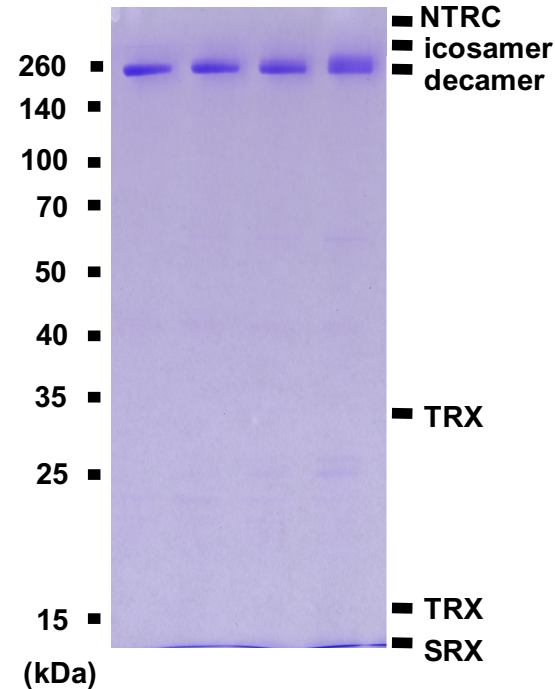

Supplement: Supplementary file 13 [file LSA-2020-00775_SdataFS9.pdf]

**A**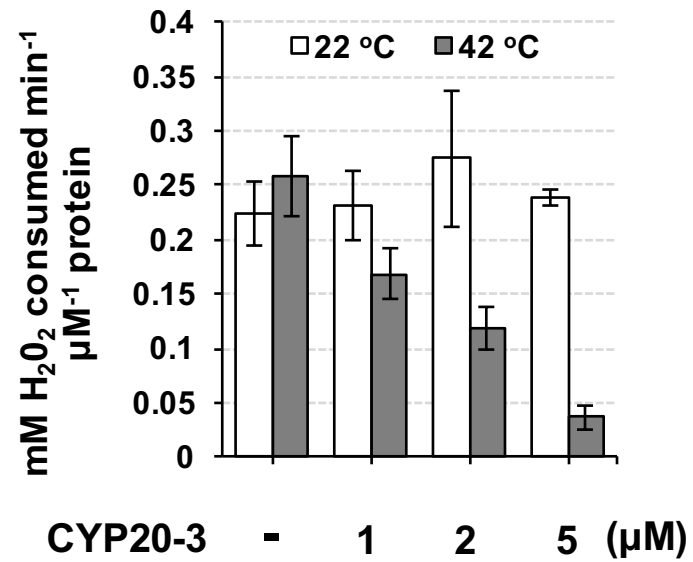**C**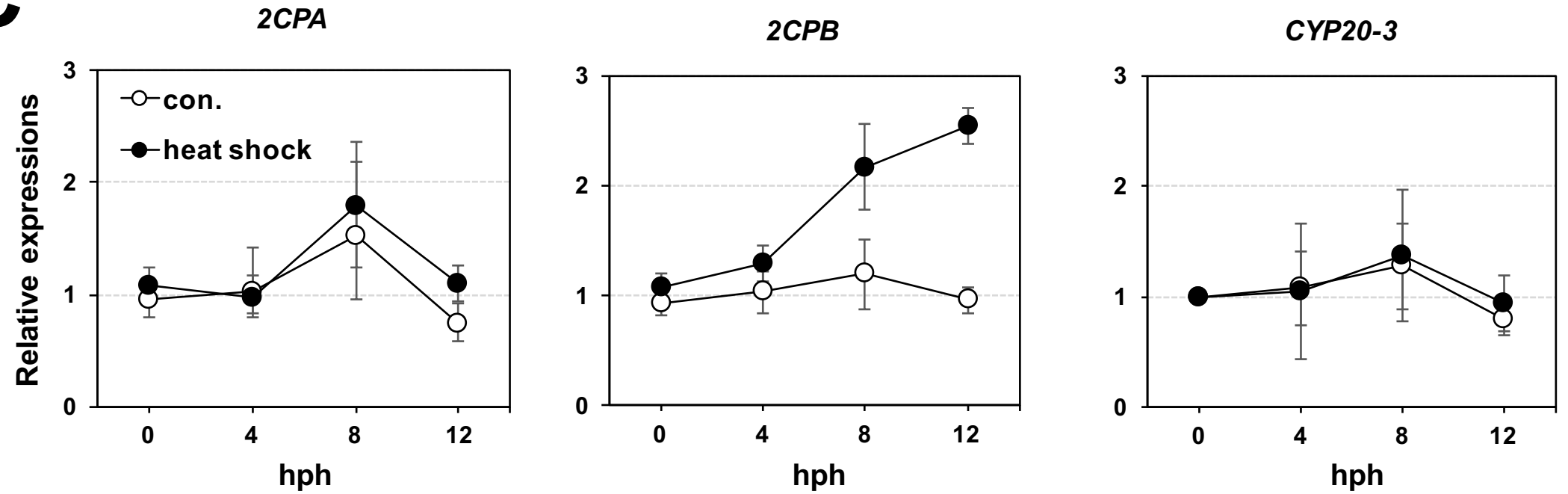**B**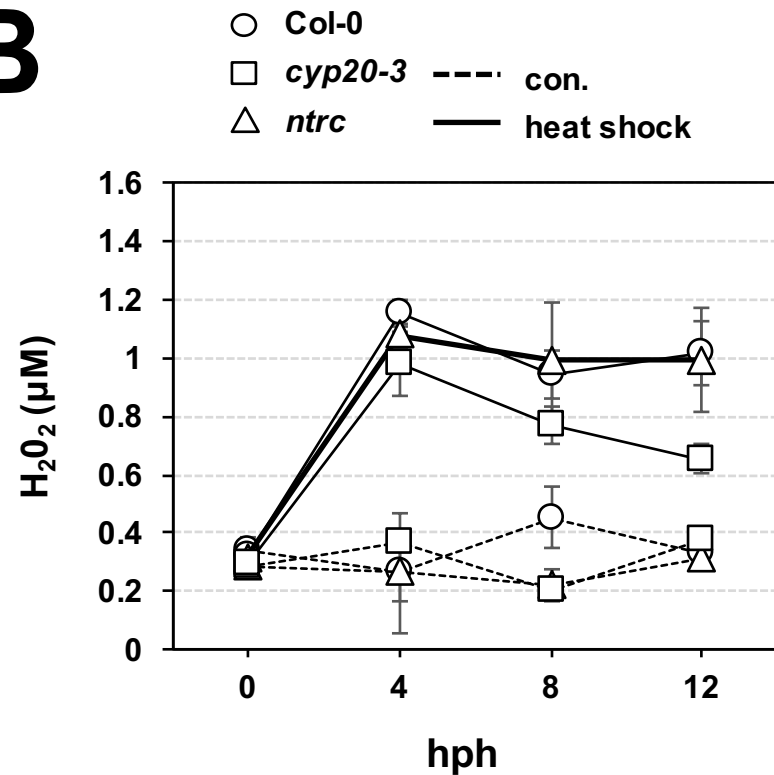

▲ *2cpa1*  
 ◆ *2cpa11*  
 ● *2cpb*  
 ■ *2cpa1/2cpb*

---- con.  
 — heat shock

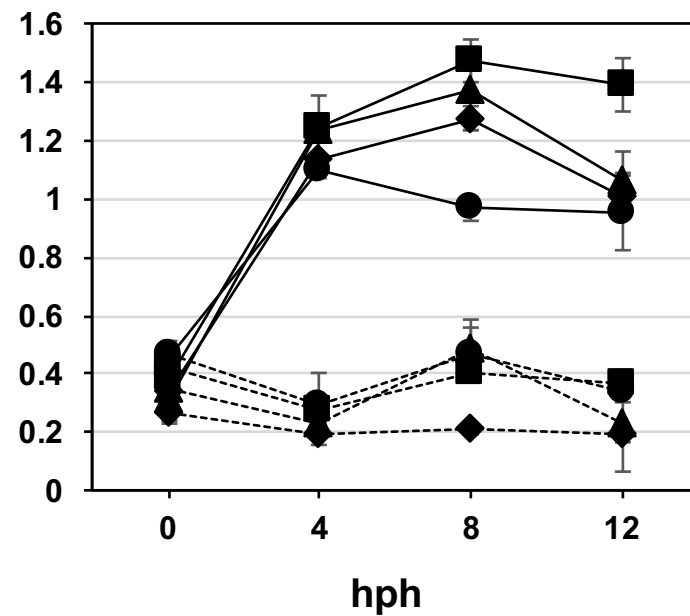**D**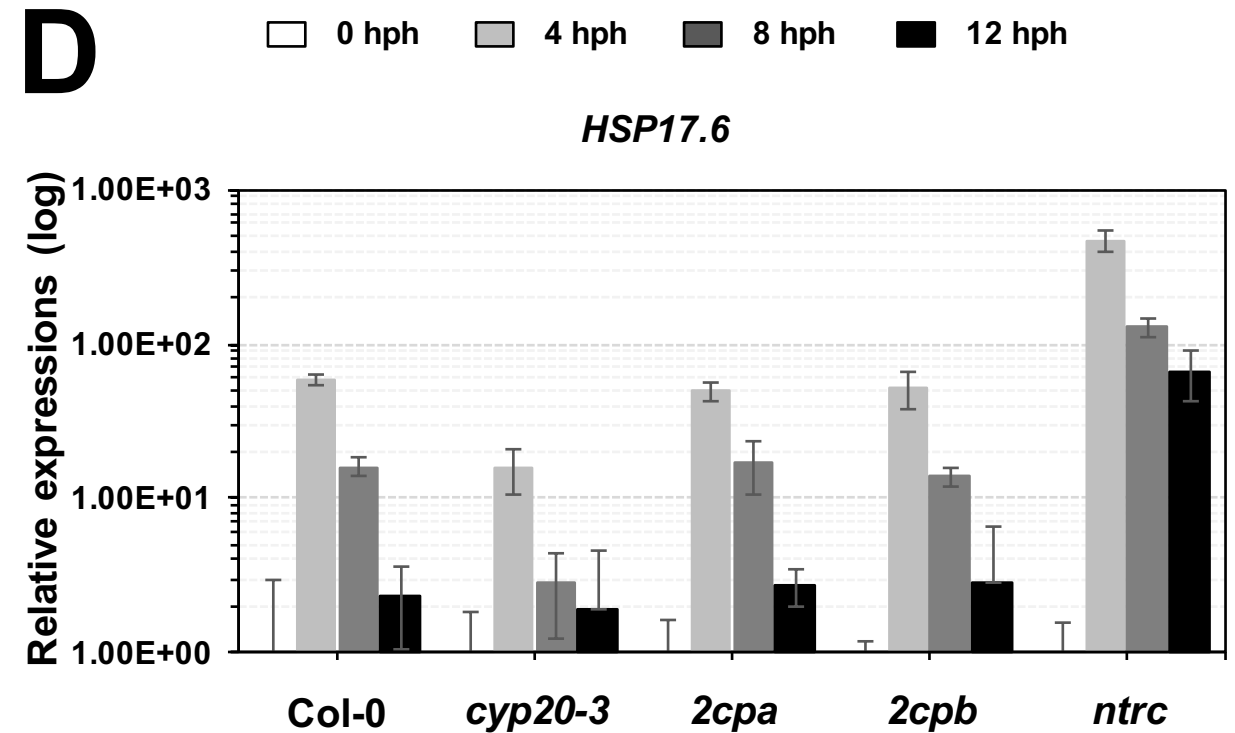

Supplement: Supplementary file 14 [file LSA-2020-00775_SdataF5.pdf]

0 hph

4 hph

8 hph

12 hph

*CYP81D11*

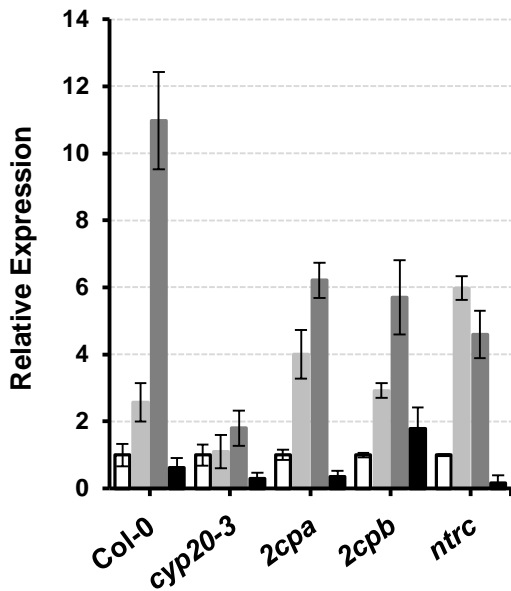

*HSP70*

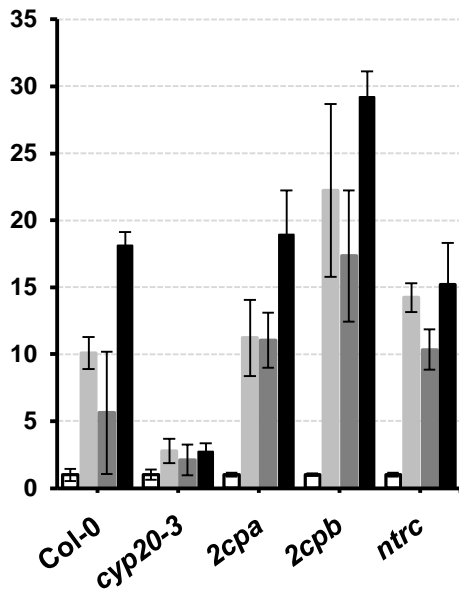

Supplement: Supplementary file 15 [file LSA-2020-00775_SdataFS10.pdf]
