# Supplementary material for: CYP20-3 deglutathionylates 2-CysPRX A and suppresses peroxide detoxification during heat stress
Source: Life Sci Alliance. 2020 Jul 30;3(9):e202000775. doi: 10.26508/lsa.202000775 (PMC7409537; doi:10.26508/lsa.202000775)
Supplement: Supplementary file 5 [file LSA-2020-00775_SdataFS4.pdf]

```
2CPA      AQADDLPLVGNKAPDFEAEAVFDQEFIKVKLSDYIGKKYVILFFYPLDFTFVCPTEITAF  60
2CPB      AQADDLPLVGNKAPDFEAEAVFDQEFIKVKLSEYIGKKYVILFFYPLDFTFVCPTEITAF  60
          ********************************:***************************

2CPA      SDRHSEFEKLNTEVLGVSVDSVFSHLAWVQTDRKSGGLGDLNYPLISDVTKSISKSFGVL  120
2CPB      SDRYEEFEKLNTEVLGVSVDSVFSHLAWVQTDRKSGGLGDLNYPLVSDITKSISKSFGVL  120
          ***:.****************************************:**:***********

2CPA      IHDQGIALRGLFIIDKEGVIQHSTINNLGIGRSVDETMRTLQALQYIQENPDEVCPAGWK  180
2CPB      IPDQGIALRGLFIIDKEGVIQHSTINNLGIGRSVDETMRTLQALQYVQENPDEVCPAGWK  180
          * ********************************************:*************

2CPA      PGEKSMKPDPKLSKEYFSAI  200
2CPB      PGEKSMKPDPKLSKEYFSAI  200
          ********************
```
