# Supplementary material for: CYP20-3 deglutathionylates 2-CysPRX A and suppresses peroxide detoxification during heat stress
Source: Life Sci Alliance. 2020 Jul 30;3(9):e202000775. doi: 10.26508/lsa.202000775 (PMC7409537; doi:10.26508/lsa.202000775)
Supplement: Supplementary file 6 [file LSA-2020-00775_SdataF2.pdf]

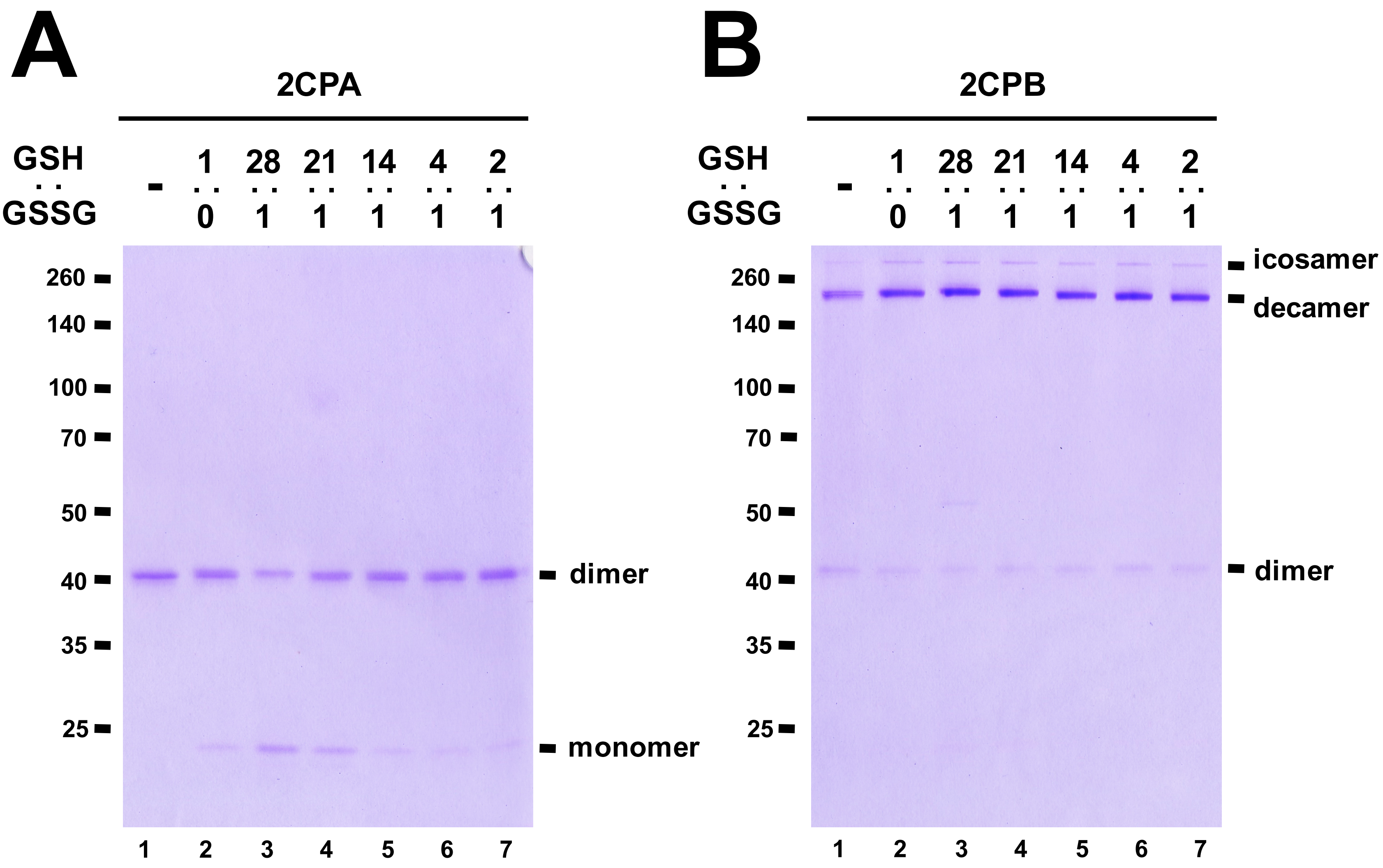
A
2CPA
GSH
GSSG
-
1 : 0
28 : 1
21 : 1
14 : 1
4 : 1
2 : 1
260
140
100
70
50
40
35
25
dimer
monomer
1 2 3 4 5 6 7
B
2CPB
GSH
GSSG
-
1 : 0
28 : 1
21 : 1
14 : 1
4 : 1
2 : 1
260
140
100
70
50
40
35
25
icosamer
decamer
dimer
1 2 3 4 5 6 7
