# Supplementary material for: CYP20-3 deglutathionylates 2-CysPRX A and suppresses peroxide detoxification during heat stress
Source: Life Sci Alliance. 2020 Jul 30;3(9):e202000775. doi: 10.26508/lsa.202000775 (PMC7409537; doi:10.26508/lsa.202000775)
Supplement: Supplementary file 16 [file LSA-2020-00775_TableS1.docx]

Table S1. Oligonucleotides used in the study.

| Name | Direction | Sequence, 5’ to 3’ | purpose |
| --- | --- | --- | --- |
| *2CPB*∙BamHI | Forward | TCGATCGGATCCGCTCAGGCTGATGATTTACCAC | Pla. cons. |
| *2CPB*∙HindIII | Reverse | GATCTGAAGCTTCTAGATAGCTGAAAAGTATTC | Pla. cons. |
| *NTRC*∙BamHI | Forward | CAAGGATCCATGGCTGCGTCTCCCA | Pla. cons. |
| *NTRC*∙HindIII | Reverse | CTTAAGCTTTCATTTATTGGCCTCA | Pla. cons. |
| *SRX*∙BamHI | Forward | TCGATCGGATCCGGAGGATCTAGCGGCGGTGTA | Pla. cons. |
| *SRX*∙NotI | Reverse | GATCTGGCGGCCGCTCAGCGAAGATGATGCCTGA | Pla. cons. |
| *2CPA*∙*Nde*I | Forward | TCGATCCATATGGCCCAGGCCGATGATCTTCCAC | Pla. cons. |
| *2CPA*∙*Hind*III | Reverse | GATCTGAAGCTTCTAAATAGCTGAGAAGTACTC | Pla. cons. |
| *2CPB*∙*Nde*I | Forward | TCGATCCATATGGCTCAGGCTGATGATTTACCAC | Pla. cons. |
| *2CPB*∙*Hind*III | Reverse | GATCTGAAGCTTCTAGATAGCTGAAAAGTATTC | Pla. cons. |
| *2CPB*∙E33D | Forward | TTCATAAAGGTGAAGCTCTCTGATTACATTGGCAAAAAGTATGTTAT | SD mutage. |
| *2CPB*∙E33D | Reverse | ATAACATACTTTTTGCCAATGTAATCAGAGAGCTTCACCTTTATGAA | SD mutage. |
| *2CPB*∙Y64H/E65S | Forward | CTGAGATTACTGCCTTCAGTGACCGTCATTCAGAATTTGAGAAGCTAAACAC | SD mutage. |
| *2CPB*∙Y64H/E65S | Reverse | GTGTTTAGCTTCTCAAATTCTGAATGACGGTCACTGAAGGCAGTAATCTCAGT | SD mutage. |
| *2CPB*∙V106I/I109V | Forward | GATTTTGAAATGGATTTAGTGACATCCGAAATAAGAGGATAATTCAGATCACCGAG | SD mutage. |
| *2CPB*∙V106I/I109V | Reverse | CTCGGTGATCTGAATTATCCTCTTATTTCGGATGTCACTAAATCCATTTCAAAATC | SD mutage. |
| *2CPB*∙P122H | Forward | GTGCAATGCCCTGATCATGGATGAGCACTCCAAAC | SD mutage. |
| *2CPB*∙P122H | Reverse | GTTTGGAGTGCTCATCCATGATCAGGGCATTGCAC | SD mutage. |
| *2CPB*∙V167I | Forward | CATCCGGGTTTTCTTGAATATACTGTAATGCCTGGAGG | SD mutage. |
| *2CPB*∙V167I | Reverse | CCTCCAGGCATTACAGTATATTCAAGAAAACCCGGATG | SD mutage. |
| *2CPA*∙I106V/V109I | Forward | CTTGGTGATCTGAACTATCCCCTTGTTTCAGATATCACTAAATCAATCTCAAAGTC | SD mutage. |
| *2CPA*∙I106V/V109I | Reverse | GACTTTGAGATTGATTTAGTGATATCTGAAACAAGGGGATAGTTCAGATCACCAAG | SD mutage. |
| *2CPA*∙Fw | Forward | AACTACTCTCATCTCTTCTCC | qRT-PCR |
| *2CPA*∙Rev | Reverse | AGGGAAGAGCGTCGAGCAAAT | qRT-PCR |
| *2CPB*∙Fw | Forward | TTCTTCCACCACCCTACT | qRT-PCR |
| *2CPB*∙Rev | Reverse | GGAACCGAGACTGGAGAAC | qRT-PCR |
| *CYP20-3*∙Fw | Forward | TAAGTCGCAATTAGTTTC | qRT-PCR |
| *CYP20-3*∙Rev | Reverse | CTAACCATGAAGT^CTTG | qRT-PCR |
| *NTRC*∙Fw | Forward | TATGCAAGAT^AGAGTGATCA | qRT-PCR |
| *NTRC*∙Rev | Reverse | ACTGTTTGGCGAATGCCCTA | qRT-PCR |
| *HSP17.6*∙Fw | Forward | CTTGCCTGGATTGAAGAAGG | qRT-PCR |
| *HSP17.6*∙Rev | Reverse | CATCGCAGCCTTAACCTGAT | qRT-PCR |
| *HSP70*∙Fw | Forward | GGAAAGTTCGAGCTCAGTGG | qRT-PCR |
| *HSP70*∙Rev | Reverse | ACCTTCCCTTGTCGTTTGTG | qRT-PCR |
| *CYP81D11*∙Fw | Forward | TCTCAACATG^GGTTTGTGAA | qRT-PCR |
| *CYP81D11*∙Rev | Reverse | AAGTATC^ATAACAAGTATGA | qRT-PCR |
| *UBC∙*Fw | Forward | CTGCGACTCAG^GGAATCTTCTAA | qRT-PCR |
| *UBC∙*Rev | Reverse | TTGTGCCATTGAATTGAACCC | qRT-PCR |
| *GAPDH∙*Fw | Forward | TTGGTGACAACAGGTCAAGCA | qRT-PCR |
| *GAPDH∙*Rev | Reverse | AAACTTGTCGCTCAATGCAATC | qRT-PCR |
| *PP2A∙*Fw | Forward | TATCGGATGACGATTCTTCGTGCAG | qRT-PCR |
| *PP2A∙*Rev | Reverse | GCTTGGTCGACTATCGGAATGAGAG | qRT-PCR |

Pla. cons.; Plasmid construction.

SD mutage.; Site-directed mutagenesis.

Restriction sites used in plasmid construction are underlined.

^^^ Position of an exon-exon junction.
